# Elevated plasma trimethyllysine is associated with incident atrial fibrillation

**DOI:** 10.1016/j.ajpc.2025.100932

**Published:** 2025-01-14

**Authors:** Mads M Svenningsson, Gard FT Svingen, Per M Ueland, Gerhard Sulo, Espen Ø Bjørnestad, Eva R Pedersen, Indu Dhar, Dennis W. Nilsen, Ottar Nygård

**Affiliations:** a Haukeland University Hospital, Department of Heart Disease, Bergen, Norway; b Bevital AS, Bergen, Norway; c University of Bergen, Department of Global Public Health and Primary Care, Bergen, Norway; d University of Bergen, Department of Clinical Science, Bergen, Norway; e Stavanger University Hospital, Department of Heart Disease, Stavanger, Norway

**Keywords:** Atrial fibrillation, Biomarkers, Trimethyllysine

## Abstract

**Background/Aim:**

Trimethyllysine (TML) is a methylated amino acid, which is linked to epigenetic regulation and can serve as a precursor of trimethylamine-N-oxide (TMAO). TMAO is a microbiota-derived metabolite and a potential risk factor of cardiovascular disease. TML has recently been linked to atherosclerosis, acute myocardial infarction and prevalent atrial fibrillation (AF). However, any association between circulating TML and incident AF has not yet been reported and was the aim of the current study in a large community based cohort.

**Methods:**

Information regarding AF was obtained by linking patient data to national health registries. Risk associations were explored by logistic regression. Potential improvements in risk reclassification were calculated by the continuous net reclassification index (NRI˃0) and the Receiver Operating Curve Area Under the Curve (ROC-AUC).

**Results:**

At baseline 3117 patients were included. During a median (25th-75th percentile) follow-up of 10.8 (9.4 – 11.2) years, 492 patients (15.8 %) developed AF. Higher plasma TML was associated with incident AF per 1 SD log-transformed TML (OR (95 % CI) 1.30 (1.16–1.46) *P* < 0.01). Further analyses also showed an increase in NRI>0 (95 % CI) of 0.24 (0.14–0.33) *P* < 0.001 and ROC-AUC (95 % CI) of 0.013 (0.004–0.022) *P* = 0.006.

**Conclusion:**

TML was associated with, and improved risk classification of, new-onset AF in this large cohort of community dwelling adults. Our results motivate further studies on the association between TML and cardiac arrhythmias.

## Graphical abstract

**Figure:**
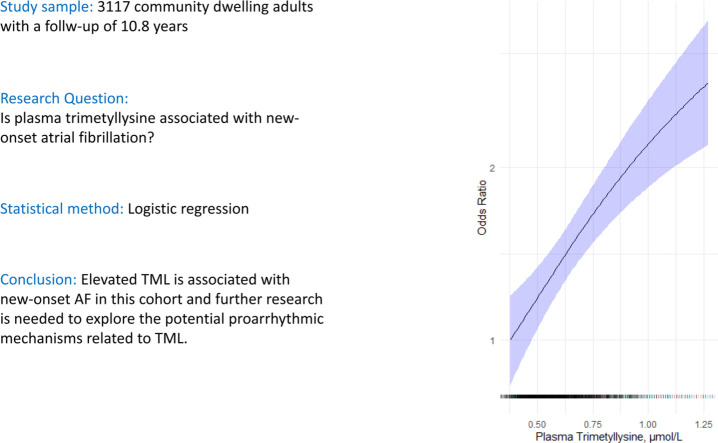

## 1 Introduction

Atrial fibrillation (AF) is a common and clinically important arrhythmia with increasing incidence and prevalence and is associated with increased morbidity, mortality, and health care costs [1]. Important risk factors for AF include male gender, higher age, smoking, diabetes mellitus (DM), hypertension (HT) and obesity. However, biomarkers have a minor role in the evaluation of AF risk [1,2]. Hence, it is important to identify new biomarkers to improve risk prediction of AF and explore potential pathophysiological mechanisms.

Trimethyllysine (TML) is a methylated amino acid, which has been linked to atherosclerosis and recently also to acute myocardial infarction (AMI) [3,4]. TML can be created endogenously from post-translational methylation (PTM) of lysine residues in proteins, such as histones, thus linking TML to epigenetic regulation. TML can also be ingested from multiple plant- and animal-derived dietary sources [5,6]. Notably, TML can serve as precursor of trimethylamine-N oxide (TMAO) a microbiome-derived metabolite that has been linked to cardiovascular disease (CVD), including incident AF [[7], [8], [9]].

Elevated plasma TML was reported in patients with prevalent AF [10], as well as related to adverse cardiovascular events among patients with stroke [11]. However, any association between circulating TML and incident AF has not yet been reported and was the aim of the current study in a large community-based cohort.

## 2 Material and methods

### 2.1 Study population and design

The study population consisted of 3317 community-dwelling individuals, born in 1925–1927, participating in the second wave of the community-based Hordaland Health Study (HUSK 2: https://husk-en.w.uib.no), with baseline examinations carried out during 1997–1999. Patients with a previous history of AF and those with missing baseline data on TML or covariates incorporated in the risk models were excluded, leaving 3117 for the final analyses.

All participants provided written informed consent. The study was conducted according to the Declaration of Helsinki, and was approved by the Regional Committee for Medical and Health Research Ethics, Norwegian Health Region West (Approval number 2015/876)

### 2.2 Clinical and biochemical data

Baseline information regarding demographics, anthropometry, anamnestic data and lifestyle were collected by questionnaires and by physical examination by study personnel, as previously described [12].

Blood samples were collected at the baseline survey and routine laboratory analyses were carried out on fresh blood samples. Study-specific analyses were performed at the laboratory of BEVITAL AS, Bergen, Norway (bevital.no) on samples stored at −80 ° C and later thawed. Plasma TML was analyzed by liquid chromatography-tandem mass spectrometry [13].

### 2.3 Endpoint data

The endpoint was defined as receiving a diagnosis of AF during hospitalization (according to the International Classification of Diseases 10th edition I48) during hospitalization or death due to AF throughout 31 December 2009. Endpoint data were collected from the Cardiovascular Disease in Norway project (CVDNOR; https://cvdnor.b.uib.no), which provides data on CVD diagnoses at discharge from Norwegian hospitals in the period 1994–2009. Information regarding death due to AF was obtained from the Norwegian Cause of Death Registry. Each participant was linked to endpoint data using a 11-digit personal identification number exclusive to each Norwegian resident.

### 2.4 Statistical analyses

Continuous and categorical variables are reported as medians (25th-75th percentiles) and counts (%), respectively. Linear trends across quartiles of plasma TML were tested by median linear regression for continuous variables, and by logistic regression for categorical variables. Differences between groups were tested by Mann-Whitney U test and Chi square test for continuous and categorical variables, respectively. Because of the potentially strong relationship between plasma TML and TMAO, we examined this correlation by calculating the Spearman's correlation coefficient, as well as the variance inflation factor to check for collinearity.

Univariate and multivariate logistic regression models were used to obtain odds ratios (ORs) [95 % Confidence intervals (CIs)] between the 4th and 1st quartile of TML regarding AF risk, and to examine TML-AF association per 1 standard deviation (SD) increment in log-transformed plasma TML. Risk associations were explored unadjusted and adjusted for sex (Model 1). An extended Model 2 was created adding body mass index (BMI), DM, current smoking, creatinine, HT, TMAO, High-sensitivity C-reactive protein (hs-CRP) and neopterin to Model 1. We also created an extended Model 3 adding former MI and anamnestic heart failure (HF) to Model 2. We did not adjust for age as the cohort was selected based on birth-year. Potential unmeasured confounders were explored by calculating the E-value [14].

We created a generalized additive model (GAM) smoothed spline using the unadjusted logistic regression model to investigate possible non-linear risk relationships between log-transformed plasma TML as a continuous variable and incident AF.

We explored reclassification by adding TML to a multivariate logistic model that included sex, HT, DM, BMI and smoking and calculated the continuous net reclassification improvement (NRI>0) and the Receiver Operating Curve Area Under the Curve (ROC-AUC).

All tests were two-sided and the significance level was set to 0.05. We used IBM SPSS Statistics for Windows, Version 23.0. Armonk, NY:IBM Corp. and R for Windows (R Core Team 2016, Vienna, Austria), utilizing the packages #survival, #Hmisc. #PredictABEL, #ROCR and #ICC.

## 3 Results

### 3.1 Baseline characteristics

Baseline characteristics according to plasma TML quartiles are shown in Table 1. Higher levels of TML were associated with male gender, and an overall more adverse CVD risk profile, including more often established coronary and cerebrovascular disease, hypertension, diabetes and higher serum creatinine, hs-CRP and neopterin, as well as usage of betablockers, statins, ACE inhibitors and angiotensin II receptor blockers. Current smoking and total cholesterol showed an inversed relationship with TML. Spearman's correlation coefficient between TML and TMAO was 0.45 (*P* < 0.001), which indicates a moderate correlation and a variance inflation factor of 1.2, ruling out collinearity.

**Table 1:** Baseline characteristics according to plasma trimethyllysine quartiles among HUSK participants.

	Plasma TML quartiles					
	N*	1	2	3	4	P*_trend_*
Plasma TML, µmol/L	3117	0.45 (0.41–0.47)	0.54 (0.52–0.56)	0.65 (0.62–0.68)	0.86 (0.78–1.02)	–
Plasma TMAO, µmol/L	3117	4.46 (3.27–6.74)	5.62 (3.68–8.40)	6.81 (4.50–10.1)	11.3 (6.26–24.2)	< 0.001
Age, years	3117	72	72	72	72	–
BMI, kg/m^2^	3117	25.0 (22.7–28.0)	25.6 (23.3–28.4)	25.8 (23.6–28.1)	26.7 (24.4–28.7)	< 0.001
Male gender, % (n)	3117	17.2 (134)	35.8 (279)	57.3 (446)	64.6 (505)	< 0.001
Current smoking, % (n)	3117	17.5 (136)	18.1 (141)	14.0 (109)	11.9 (93)	< 0.001
Diabetes, % (n)	3117	5.5 (43)	5.0 (39)	7.7 (60)	8.4 (66)	0.004
Hypertension, % (n)	3117	22.4 (174)	25.9 (202)	29.9 (233)	35.9 (281)	< 0.001
Betablockers, % (n)	3117	17.1 (133)	20.3 (158)	23.1 (180)	26.2 (205)	< 0.001
Statins, % (n)	3117	9.5 (74)	9.8 (76)	12.3 (96)	13.3 (104)	0.006
ACEi or ARB, % (n)	3117	8.8 (68)	12.2 (95)	12.3 (96)	18.4 (144)	< 0.001
Serum lipid parameters						
Total cholesterol, mmol/L	3117	6.47 (5.72–7.21)	6.33 (5.63–7.04)	6.13 (5.45–6.94)	6.01 (5.24–6.81)	< 0.001
HDL-C, mmol/L	3117	1.42 (1.22–1.71)	1.35 (1.13–1.62)	1.26 (1.03–1.53)	1.20 (0.98–1.43)	< 0.001
Triglycerides, mmol/L	3117	1.45 (1.08–2.04)	1.60 (1.18–2.12)	1.63 (1.17–2.25)	1.71 (1.27–2.43)	< 0.001
Serum Creatinine µmol/L	3117	82 (76–88)	88 (82–95)	95 (87–104)	102 (92–114)	< 0.001
Plasma/serum methionine cycle metabolites						
Homocysteine, µmol/L	3112	10.9 (9.2–13.0)	11.9 (10.0–14.2)	12.7 (10.8–14.9)	13.4 (11.4–16.3)	< 0.001
Cysteine, µmol/L	3112	300 (282–317)	305 (289–322)	307 (291–325)	311 (293–329)	< 0.001
Betaine, µmol/L	3117	34.3 (28.8–41.6)	36.9 (31.5–43.5)	38.3 (32.6–44.2)	38.5 (32.9–45.8)	< 0.001
Plasma/serum markers of inflammation						
CRP, mg/L	3031	1.38 (0.60–2.75)	1.56 (0.74–3.15)	1.51 (0.72–3.00)	1.70 (0.78–3.18)	0.008
Neopterin, nmol/L	3107	8.05 (6.82–9.75)	8.48 (7.31–10.1)	8.92 (7.29–11.07)	9.27 (7.73–11.7)	< 0.001
Anamnestic MI, % (n)	3095	4.7 (36)	7.7 (60)	11.3 (87)	13.9 (109)	< 0.001
Anamnestic angina, % (n)	3117	7.8 (60)	10.7 (83)	13.5 (105)	12.9 (101)	< 0.001
Anamnestic stroke, % (n)	3117	3.3 (26)	3.7 (29)	3.6 (28)	6.0 (47)	0.013
High alcohol intake, % (n)*	2079	1.2 (9)	4.1 (32)	5.9 (46)	2.7 (21)	0.03

ACEi, Angiotensin-converting enzyme inhibitors; ARB, angiotensine reseptor blockers; BMI, body mass index; CRP, C-reactive-protein; HDL-C, high density lipoprotein cholesterol; MI, myocardial infarction; TMAO, Trimethylamine N-oxide; TML, trimethyllysine.

⁎ > 16 units per two weeks.

### 3.2 The association between plasma TML and incident atrial fibrillation

**Fig. 1:**
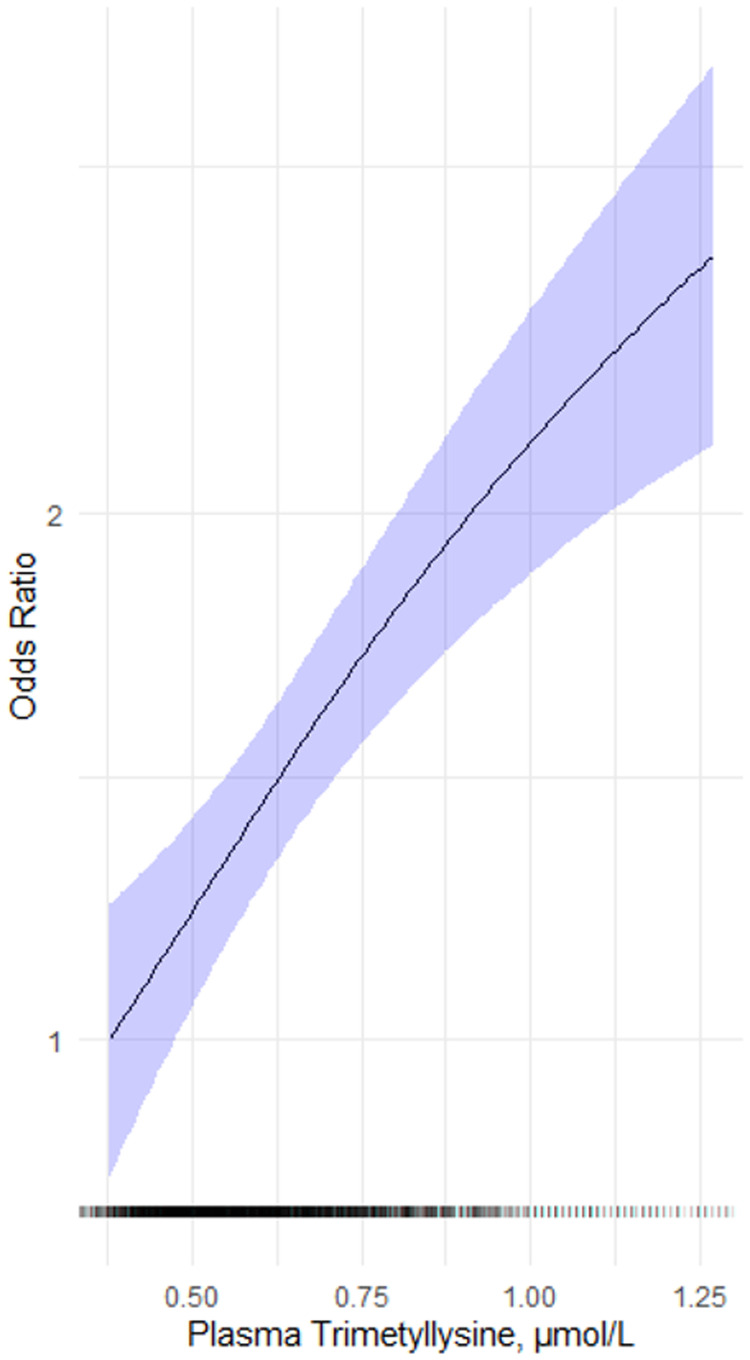
The unadjusted risk relationship using a generalized additive model between plasma TML and new-onset atrial fibrillation in HUSK. The shaded area covering the spline depicts the 95 % confidence interval. A density plot is superimposed along the x-axis. The x-axis is truncated, excluding the lower and upper 2.5 %.

For the prospective analyses 3117 patients were included. During a median (25th-75th percentile) follow-up of 10.8 (9.4 – 11.2) years 492 patients (15.8 %) developed AF. Baseline median (25th-75th percentile) TML was higher among those who received a diagnosis of AF (0.64 (0.49 – 0.71) µmol/L) than those who did not median (0.58 (0.53 – 0.79) µmol/L), *P* < 0.001. Accordingly, higher plasma TML was also associated with incident AF in Model 2 and 3 (Table 2) (OR (95 % CI) per 1 SD 1.30 (1.16–1.47) *P* < 0.01), and the association was essentially linear (Fig. 1).

**Table 2:** Risk associations between TML and AF.

	Unadjusted		Model 1#		Model 2*		Model 3†	
	OR (95 % CI)	*P-*Value	OR (95 % CI)	*P-*Value	OR (95 % CI)	*P-*Value	OR (95 % CI)	*P-*Value
Plasma TML								
2nd vs 1st quartile	1.06 (0.78–1.43)	*P* = 7.1	0.97 (0.71–1.31)	*P* = 0.82	0.94 (0.68 – 1.29)	*P* = 0.69	0.90 (0.65–1.24)	*P* = 0.51
3rd vs 1st quartile	1.61 (1.21–2.14)	*P* < 0.01	1.33 (0.99–1.80)	*P* = 0.06	1.38 (1.01–1.88)	*P* = 0.05	1.32 (0.96–1.81)	*P* = 0.08
4th vs 1st quartile	1.91 (1.45–2.52)	*P* < 0.01	1.53 (1.14–2.06)	*P* < 0.01	1.62 (1.15–2.28)	*P* < 0.01	1.56 (1.10–2.21)	*P* = 0.01
Per 1 SD	1.33 (1.22–1.45)	*P* < 0.01	1.25 (1.14–1.38)	*P* < 0.01	1.32 (1.17–1.49)	*P* < 0.01	1.31 (1.16–1.48)	*P* < 0.01

OR indicates odds ratio; CI, confidence interval; values per 1 SD log transformed; SD, standard deviation.

# adjusted for sex.

⁎ adjusted for sex, BMI, DM, current smoking, creatinine, HT, TMAO, CRP and neopterin.

† adjusted for sex, BMI, DM, current smoking, creatinine, HT, TMAO, CRP, neopterin, former MI and anamnestic HF.

Application of the E-value formula to the multivariate model revealed E-values for the OR and lower CI of 2.47 and 1.43, respectively.

### 3.3 Reclassification and model discrimination

When adding TML to a logistic regression model containing sex, BMI, smoking, hypertension, diabetes and TMAO the NRI>0 (95 % CI) was 0.24 (0.14–0.33) *P* < 0.001. Further ROC analyses also showed an increase in area under the curve from 0.613 to 0.626 with an area difference (95 % CI) of 0.013 (0.004–0.022) *P* = 0.006.

## 4 Discussion

### 4.1 Principal findings

In this large cohort of community-dwelling adults higher plasma levels of TML were associated with increased risk of developing AF during long-term follow-up. TML also improved reclassification of individuals beyond traditional risk factors.

### 4.2 TML and the association with AF and CVD in previous studies

To our knowledge, our study is the first to report on the association between TML and incident AF.

Data on TML and CVD is scarce, although some studies have shed light on the association. In an untargeted metabolomics study TML was identified as a predictor of incident CVD-risk, and TML has been shown to predict CVD mortality [15,16]. Moreover, TML is associated with acute myocardial infarction in patients with suspected stable coronary artery disease and with acute coronary syndrome among patients admitted to the emergency department due to chest pain [3,17]. Furthermore, TML has been shown to correlate with existing HF, and higher levels of TML is associated with a worse prognosis [18]. Regarding AF, one earlier study examining TML among patients with ischemic stroke (IS) found that TML was elevated among patients with both IS and prevalent AF whereas, patients with isolated IS had a lower plasma TML concentration compared to healthy controls [10].

The association between TML and AF may thus be mediated through other cardiovascular diseases; however adjusting for former myocardial infarction and anamnestic HF did not alter the risk estimates in this relatively healthy population.

### 4.3 TML, epigenetic regulation and inflammation

TML can be created by PTM of lysine. In general, PTM of proteins alters cellular processes by affecting protein properties. Notably, the interaction between methylated lysine residues and histones is considered as a key regulator of dynamic epigenetic processes and cellular maintenance offering several potential links between TML and AF. First, TML is a precursor of carnitine synthesis. Disturbances in carnitine homeostasis have been linked to reduced nitric oxide signaling and endothelial function, suggesting a possible link between TML and AF [19,20]. Second, TML and histone methylation is associated with pathways associated with coronary artery disease, hypertension, cardiac hypertrophy, and endothelial dysfunction, in mice and humans, which again recently has been linked to AF [19,[21], [22], [23], [24]]. Third, histone methylation is shown to be central in regulation of metalloproteinases (MMPs), via inflammation and oxidative stress. MMPs play a key role in maintaining the extra cellular matrix (ECM), which in turn have been shown to play a role in the development of CVD in general and particularly AF [2,25]. In the present cohort TML was positively associated with hs-CRP and neopterin; however, adjusting for these inflammatory markers did not alter the risk estimates significantly. Thus, the link between TML and AF could be caused by electrophysiologic and morphologic atrial changes due to fibrosis and inflammation.

### 4.4 TML, gut-microbiota and increased body weight

Gut-microbial metabolites have been suggested as a potential paradigm shift for identifying predictive biomarkers, cardiovascular risk stratification and future treatment options [26]. Any association between TML and AF could be confounded by the previous reported positive relationship between plasma TMAO and incident AF [8]. However, including TMAO in model 2 did not alter the risk estimates. Accordingly, dietary TML has not shown to increase circulating TMAO in mice [15]. TML is also associated with other known risk factors for AF including increased body weight, incident type 2 diabetes and altered gut microbiome among individuals with and without insulin resistance [27,28]. However, adjusting for BMI and diabetes did not alter the risk estimates. Taken together, this suggests that TML exerts effects independent of TMAO and the known link with increased body weight and diabetes.

## 5 Strengths and limitations

Key strengths of this study include the large sample size, the long-term follow-up, its prospective design, as well as endpoints obtained from national health registries. However, our study has limitations. First, we only had one single measurement of plasma TML available, which may give rise to regression dilution bias. Second, as with any observational study there is a possibility of residual confounding. However, we calculated an E-value indicating that such a confounder would have to be associated with both TML and AF with a strength of RR > 1.43. We are not aware of any potential confounder of this strength, conditional on the variables included in the multivariate model. Third, while TML did improve patient reclassification and model discrimination, it is uncertain if this information will confer any clinical benefit regarding risk stratification. Fourth, the cohort consisted primarily of Caucasian individuals from Western Norway and the results need to be replicated in populations with different ethnicity and demographics.

## 6 Conclusion

Elevated TML was associated with increased risk of new-onset AF in this large cohort of community dwelling adults. Further research is needed to explore the potential proarrhythmic mechanisms related to TML.

## CRediT authorship contribution statement

**Mads M Svenningsson:** Writing – original draft, Methodology, Formal analysis. **Gard FT Svingen:** Writing – original draft, Methodology, Formal analysis. **Per M Ueland:** Writing – review & editing. **Gerhard Sulo:** Writing – review & editing, Validation, Investigation. **Espen Ø Bjørnestad:** Writing – review & editing. **Eva R Pedersen:** Writing – review & editing. **Indu Dhar:** Writing – review & editing. **Dennis W. Nilsen:** Writing – review & editing, Investigation. **Ottar Nygård:** Writing – review & editing, Supervision, Project administration, Investigation, Conceptualization.

## Declaration of competing interest

The authors declare the following financial interests/personal relationships which may be considered as potential competing interests:

Ottar Nygaard reports financial support was provided by Statens Helseundersøkelser. Ottar Nygaard reports financial support was provided by Stiftelsen for forskning på funksjonell vitamin B12-mangel. Ottar Nygaard reports financial support was provided by The research council of Norway. If there are other authors, they declare that they have no known competing financial interests or personal relationships that could have appeared to influence the work reported in this paper.
